# Recombinant shrimp antimicrobial peptides as alternative biotherapeutics in aquaculture: An exploration of future prospects

**DOI:** 10.1016/j.cirep.2026.200271

**Published:** 2026-02-09

**Authors:** Belman Ananya, Vijay Gundmi Apurva, Indrani Karunasagar, Anirban Chakraborty, Biswajit Maiti

**Affiliations:** a Nitte (Deemed to be University), Nitte University Centre for Science Education and Research (NUCSER), Department of Bio & Nano Technology, Paneer Campus, Deralakatte, Mangalore, 575018, India; b Nitte (Deemed to be University), DST Technology Enabling Centre, FAO Reference Centre for AMR and Aquaculture Biosecurity, Paneer Campus, Deralakatte, Mangalore, 575018, India; c Nitte (Deemed to be University), Nitte University Centre for Science Education and Research (NUCSER), Department of Molecular Genetics and Cancer, Paneer Campus, Deralakatte, Mangalore, 575018, India

**Keywords:** Antimicrobial peptides, Aquaculture, Recombinant AMPs, Shrimp farming, Shrimp disease

## Abstract

Multifaceted disease problems plague shrimp farming on an epic scale, resulting in colossal economic losses and sustainability issues. The overuse and misuse of antibiotics in management of disease outbreaks is a growing concern because of the emergence of antibiotic resistance in pathogens. This necessitates more sustainable and innovative solutions. Several alternatives are under study, and some have already been found suitable for use in shrimp hatcheries and aquaculture farms. Antimicrobial peptides (AMPs) could be viable, environmentally acceptable biotherapeutic agents to address the issue of antibiotic resistance. Advancements in studies on the expression profiling of AMPs in shrimp and their synthesis through genetic engineering approaches strongly indicate their potential for application in aquaculture systems. In recent years, there have been conspicuous efforts to enhance the stability of AMP and to facilitate targeted delivery in field conditions. The future of AMP research will focus on developing AMP nanoformulations to enhance bioavailability and on host genome editing to improve crop adaptability to potential pathogens. Thus, AMP-based solutions could be integrated to transform disease management strategies in farmed shrimp and to steer the industry towards economic sustainability. The use of AMPs as alternatives to antibiotics addresses antibiotic use related resistance and residues and is likely to contribute to better environmental health. It also promises profitability and long-term sustainability of the sector through healthy shrimp production. This review presents a comprehensive information on shrimp AMP types, their mechanisms of action, and their efficacy in combating pathogen-induced diseases in shrimp farming.

## Highlights

• Shrimp aquaculture experiences major economic setbacks due to various disease outbreaks.
• Misuse of antibiotics in shrimp farming has led to widespread antibiotic resistance.
• AMPs emerge as safe and sustainable alternatives to conventional antibiotics.
• Recombinant AMPs can ensure consistent, scalable production for efficient application.
• Biotechnology and nanoformulation of AMPs can significantly improve their stability, bioavailability, and therapeutic efficacy.

## Introduction

The oceanic expanses, which cover approximately 71 % of the Earth’s surface, serve as an indispensable source of aquatic food and a means of sustenance. It plays a major role in addressing global food security by providing an ample and diverse array of seafood rich in protein and other essential nutrients. Since wild-caught fish from capture fisheries are dwindling, cultured fish are necessary to meet the increasing demand, enabled by high-yield aquaculture in limited space [1]. Intensive and semi-intensive shrimp culture, also known as shrimp farming, practiced in the coastal waters of tropical regions worldwide, is one of the most widely pursued aquaculture practices. This involves the controlled cultivation and breeding of shrimp for commercial purposes. Shrimp cultivation has successfully established itself as a significant revenue-generating sector, owing to the high demand for shrimp in global markets, contributing to the foreign exchange reserves of low- and middle-income countries and providing livelihoods for many living in coastal areas.

One of the major challenges in shrimp culture is disease outbreaks, primarily caused by viruses and bacteria, which can lead to mass mortality. These outbreaks also adversely affect the socioeconomic sector, specifically the economy and employment [2,3]. The need of the hour is to develop effective disease prevention and management practices to ensure the long-term viability and economic profitability of the sector [4]. The most common method for controlling disease outbreaks or infections in shrimp aquaculture has been the empirical use of antibiotics [5]. Not just pathogens; the impact has also affected innocuous organisms in the culture environment, which participate in biogeochemical cycles. The proliferation of antibiotic-resistant bacteria over time represents a major obstacle to the effectiveness of antibiotics in disease management [6].

Recently, there has been increasing attention to developing alternatives for sustainable aquaculture use. One such alternative is the antimicrobial peptide (AMP), a biologically active small molecule that exhibits potent antimicrobial properties. They are known to be crucial innate immune effectors in various crustaceans due to their activity against disease-causing microbes [7]. In the aquaculture industry, AMPs hold promise as next-generation bioactive compounds for improving shrimp health and overall growth performance [8]. Recombinant AMP-enriched feeds could offer cost-efficient and large-scale implementation in aquaculture. Their inclusion in farming practices may improve product quality for international markets [9,10]. However, their field application in shrimp aquaculture to mitigate pathogenic microorganisms remains largely unexplored necessitating in-depth studies of potential challenges such as field efficacy, stability, effective delivery system, cost-effective production, and safety [11].

**Fig. 1:**
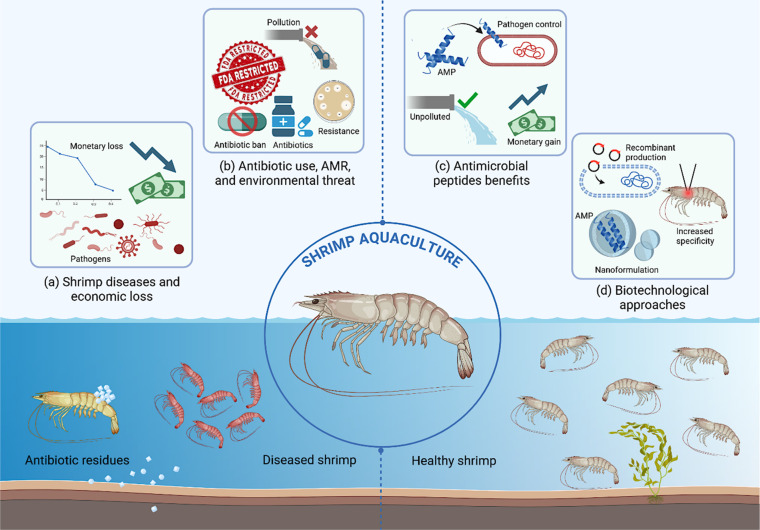
Improving shrimp farming sustainability with antimicrobial peptides and biotechnological approaches: (a) Shrimp diseases and economic loss: Detrimental effects of diseases on shrimp health and aquaculture profitability, highlighting how pathogens can cause significant financial losses in shrimp farming. (b) Antibiotic use, antimicrobial resistance (AMR), and environmental threat: The consequences of using banned antibiotics to control diseases that lead to AMR and environmental contamination due to antibiotic residues. (c) Antimicrobial peptides (AMPs) benefits: AMPs as a promising alternative to antibiotics show effective pathogen control without giving rise to AMR, improve shrimp health, enhance monetary benefits in the shrimp industry, and support improved environmental health. (d) Biotechnological approaches: Innovative genetic and biotechnological approaches such as recombinant production of AMPs, and their nanoformulation, help enhance the shrimp disease control with increased specificity.

This review details the importance of AMPs in managing and mitigating diseases in shrimp aquaculture. It presents a comprehensive assessment of the current state of shrimp AMP research, including their classification systems, their mechanisms, and efficacy against various shrimp pathogens. Furthermore, strategies that can potentially enhance the practicality of shrimp AMPs in aquaculture settings, such as nanoformulation, which includes incorporating AMPs into nanoparticles, aiming to enhance their stability and specificity in bioavailability are discussed. The reliance on antibiotics can be mitigated by integrating AMPs as an environmentally friendly measure to promote the long-term productivity and sustainability of the shrimp aquaculture sector (Fig. 1).

## Challenges in shrimp farming: economic instability and disease outbreaks

Aquaculture is recognized globally as a sustainable alternative that can supplement the demand deficit from capture fisheries. There has been an incremental growth in the shrimp farming sector, leading to enhanced overall productivity [12]. Shrimp farming makes a major economic contribution to aquaculture due to its high market value. From 0.6 million tonnes in 1980, the shrimp production, has crossed over 5 million tonnes in 2022, reflecting a phenomenal increase, and it is expected to reach around 7.28 million tonnes by 2025. These statistics highlight its essential role in meeting the food demand at the global level [13]. East, Southeast Asia, and Latin America are the major shrimp producers, while the highest consumption is in the United States, the European Union, and Japan. China, Thailand, India, Mexico, Malaysia, Vietnam, Indonesia, the Philippines, and Brazil are the primary producers, accounting for over 80 % of global shrimp yield [13,14]. It not only ensures food security but also provides employment, livelihoods, and economic development. Disease outbreaks are natural consequences of intensive farming and are major obstacles to sector growth [[15], [16], [17]]. The most significant outbreaks that have had a catastrophic impact are linked to the white spot syndrome virus (WSSV) and acute hepatopancreatic necrosis disease (AHPND), caused by *Vibrio parahaemolyticus* [18,19]. According to the Food and Agriculture Organization (FAO) estimates, the annual loss due to these two causative agents exceeds 9 billion US dollars, accounting for approximately 15 % of the global value of farmed fish and shellfish aquaculture [20]. In intensive and semi-intensive farming, the stress due to high stocking density, inputs, and dynamic changes in the aquatic environment, causes the rapid multiplication of opportunistic pathogens present in the shrimp pond sediment, water, animals, or feed [21,22]. WSSV, infectious hypodermal and hematopoietic necrosis virus (IHHNV), yellow head virus (YHD), infectious myonecrosis virus (IMNV), and Taura syndrome virus (TSV) are among the many viruses that cause disease in cultured shrimp [23]. Since *Vibrios* are autochthonous to the aquatic environment, vibriosis, mainly caused by *Vibrio* spp.*,* including *V. harveyi, V. alginolyticus*, and *V. parahaemolyticus*, is a major disease that poses a significant threat to shrimp culture [24]. AHPND, caused by a pathogenic strain of *V. parahaemolyticus* harbouring the PirA and PirB toxins, is a disease that has caused significant losses in the shrimp aquaculture sector [25]. It has affected shrimp production by causing a nearly 60 % decline in production in affected regions, resulting in an economic loss of 1 billion US dollars globally [26]. White faeces disease (WFD), characterized by white faeces floating in the water, is another disease that significantly affects *Penaeus vannamei,* also known as Pacific white shrimp. This disease is typically caused by various species of *Vibrio,* including *V. vulnificus, V. mimicus, V. harveyi, V. alginolyticus, V. fluvialis*, and *V. parahaemolyticus*, with mortality rates as high as 30 % [27,28]. In addition to their impact on shrimp health, *Vibrio* spp. cause food-related gastroenteritis illness in humans primarily through the consumption of raw shellfish [29].

Antimicrobial agents are indispensable options for disease control and have been used empirically without ascertaining the cause of the disease. Since viral and bacterial diseases often exhibit identical clinical signs and spread quickly in aquatic environments, antibiotics are the preferred choice [30]. Antibiotic classes like fluoroquinolones, tetracyclines, quinolones, sulfonamides, chloramphenicols, and nitrofurans are used as prophylactic and therapeutic agents in shrimp culture [31,32]. Moreover, numerous shrimp farms use antibiotics as prophylactic measures to prevent the onset of diseases. Though antibiotics are quite effective in controlling the disease, excessive use can adversely affect aquatic ecosystems, leading to a buildup of harmful residues and the emergence of antibiotic-resistant bacteria [33].

Although the Food and Drug Administration (FDA) and many other countries have banned antibiotics in farmed animals and aquaculture, antibiotic residues continue to be detected at entry checkpoints when examining imported shrimps from Southeast Asian countries [34]. Additionally, frozen seafood sources and retail establishments also show the presence of antibiotic residues, which is quite concerning [35]. The prevalence of antibiotic residues in natural water bodies receiving discharge from shrimp farms has also been reported [36]. Given that farmed shrimps are likely to be treated with antibiotics and are exported worldwide, this can contribute to the spread of antimicrobial-resistant (AMR) microorganisms. According to the World Health Organization (WHO), the spread of AMR through shrimp exports could seriously harm human health [37].

What compounds the issue is that antibiotics are used in several other food sectors, and there is a strongly possibility shrimp farm water could mix with effluents from livestock, poultry, piggery, pharmaceutical industry, hospital waste, anthropogenic effluents, agriculture etc. further increasing the load of antibiotics and AMR bacteria in shrimp farm water. Therefore, it is absolutely essential that antibiotics are completely prohibited and other alternatives be studied for disease management in aquaculture [38,39].

## Antimicrobial peptides

Due to the absence of adaptive immunity, crustaceans rely heavily on innate immune responses to defend against microbial threats and other stressors [40,41]. This robust system integrates diverse defense strategies, including microbial pattern recognition, coagulation pathways, the prophenoloxidase (proPO) cascade, hemocyte activation, and various immune responses [42]. The initiation of the proPO cascade is mediated by the recognition of pathogen-associated molecular patterns (PAMPs) by pattern-recognition proteins (PRPs), triggering a sequential serine proteinase pathway that leads to the activation of phenoloxidase (PO). Melanin synthesis and the production of reactive cytotoxic intermediates are driven by PO functioning as an effective antimicrobial barrier against invading pathogens [43,44]. It has been reported that when shrimps are challenged with pathogens, toll-like receptor (TLR) gene gets significantly upregulated, indicating the involvement of innate immunity [45]. Another study showed that thrombospondins (TSPs), which are extracellular glycoproteins involved in physiological regulation, gets downregulated post-viral infection. This strategy is likely to evade host immune responses [46]. In addition to these innate immune components, AMPs also play key roles in shrimp immunity, effectively protecting against invading pathogens [47]. Beyond the antimicrobial activity of AMPs, they are now recognized as critical elements of the immune network, with their expression being majorly controlled by the components of key signalling pathways such as Toll, immune deficiency (IMD), and Janus Kinase/Signal Transducer and Activator of Transcription (JAK-STAT), thereby connecting AMP synthesis directly to immune activation [48,49]. AMPs function in coordination with the prophenoloxidase (proPO) system, which promotes melanization and reinforces immune processes such as pathogen encapsulation and immobilization [44]. Furthermore, AMPs are closely linked to hemocyte-mediated immunity, promoting hemocyte activation and phagocytic activity, thereby enhancing cellular defense mechanisms [50]. Collectively, these findings demonstrate that AMPs function not only as direct antimicrobial agents, but also as integral mediators that coordinate the broader shrimp innate immune defense network.

As described above, AMPs are small proteins naturally produced by various life forms, that function as primary barrier against pathogenic attacks in eukaryotes. They are also produced in response to competition for prokaryote growth [51]. They possess broad-spectrum inhibitory activity against bacteria, viruses, and fungi and also demonstrate anti-tumor activity [52]. The first AMP, gramicidin, was discovered from a soil bacteria, *Brevibacillus* in 1939 [53]. This was followed by the discovery of tyrocidine in 1941, another AMP that had an antagonistic effect against various bacteria. Subsequently, another AMP, named purothionin, was isolated from a plant source and it was found to be effective against certain bacteria and fungi [54]. Defensin was the first AMP isolated from animal sources [50]. Following these early reports, several AMPs have been isolated from various bacteria, fungi, and animals. Based on statistical data from antimicrobial peptide database 3 (APD3), an AMP database, AMPs are classified by origin, including microorganisms, insects, amphibians, and humans. AMPs found in lower aquatic life forms are listed in this database [55]. Marine invertebrates, such as crustaceans, depend solely on their innate immune system, including AMPs, to combat infections, as they lack adaptive immunity [56]. Thus, shrimp are now considered a source of several potent AMPs that are highly effective against a wide range of microbial threats. The major AMPs identified in shrimp include penaeidins, crustins, anti-lipopolysaccharide factors (ALFs), stylicins, and lysozymes [[57], [58], [59]]. The shrimp immune system produces a wide array of AMPs that differ in molecular structure and pathogen specificity, resulting in varied efficacy against Gram-positive and Gram-negative pathogens in species such as *P. vannamei* and *Penaeus monodon* [60]. In a study in which a crustin isoform was tested against infections in *P. vannamei*, it was found that shrimp mortality increased after *Vibrio penaeicida* infection but not after *Fusarium oxysporum* infection, highlighting the selective role of the crustin isoform, which varied with the pathogen [61]. Yet another study in *P. monodon* has revealed that crustinPm1 selectively inhibits Gram-positive bacteria, such as *Streptococcus aureus* and *Streptococcus iniae*, whereas crustinPm4 exhibits broader antimicrobial activity targeting both Gram-positive and Gram-negative bacteria, including *Escherichia coli* and *V. harveyi*. The expression of crustin Pm4 increased notably following WSSV infection, suggesting a dual role for crustins in protecting shrimp against bacterial and viral infections [62]. In a study in which shrimp were challenged with *Vibrio* infection, most AMPs, including crustins, lysozymes, and penaeidins, showed a drop in expression during the first h post-infection (0–12 h), but an increase in the late phase (24 h). In contrast, ALFs showed early transcript elevation, suggesting a role in the initial immune response in shrimp. Such comparisons in expression patterns among AMP families suggest that each AMP plays a unique role in enhancing immune defense and supporting shrimp survival [63].

### Mechanisms of action of AMPs

The complexity and diversity of AMP mechanisms of action reflect their adaptation to a wide variety of microbial threats. AMPs adopt multiple strategies to mitigate pathogens, including cell penetration, targeting intracellular molecules, and membrane disruption [64]. Although most AMPs are characterized as membrane-binding, a few exert their effects by targeting intracellular mechanisms, such as DNA, RNA, or protein function [65]. AMPs also exhibit key physicochemical features, such as defined secondary structures and cationic charges typically ranging from +2 to +9. These contribute to their antimicrobial activity. Further, structural conformations facilitate destabilization of microbial membranes. Upon interaction with microbial membranes, α-helical AMPs undergo conformational changes that support binding and insertion into lipid bilayers [66]. Membrane permeabilization by AMPs' action can be further categorised into transmembrane pore formation and non-pore models, such as the carpet model [67]. In addition to targeting membranes, AMPs can also target other potential sites, such as the cell wall and intracellular molecules. For instance, binding AMPs to peptidoglycan (PG) precursors inhibits their synthesis, rendering the bacteria highly susceptible to death [68,69]. A shrimp-derived crustin AMP was shown to cause inhibition of Gram-positive bacteria by binding to lipoteichoic acids (LTA) and PG [57]. Additionally, the interaction of a shrimp AMP with lipopolysaccharide (LPS) and other cell wall components, such as LTA in Gram-positive bacteria, is a proposed mechanism [70]. A novel penaeidin from shrimp, which contained an unusual serine-rich region and a penaeidin domain consisting of proline-rich region and a cysteine-rich region, both of which exhibited antimicrobial activity by binding to polysaccharides has been reported [71]. In another study, the researchers used a peptide synthesized to mimic the LPS-binding domain of ALF from shrimp to analyze the relationship between AMP structure and antimicrobial activity. These results indicated high antimicrobial activity against a wide range of bacteria, and its structure was observed to play an important role in this activity [72]. In the case of WSSV viral infections, shrimp ALFPm3 bound efficiently to the virus's envelope protein to inhibit infection [73]. Recombinant AMP ALFPm3 from shrimp exhibited antimicrobial activity by binding to Lipid A. Functional variances like binding efficiencies can occur in response to differences in nucleotide sequences in AMPs [74]. The proline-rich domain in penaeidins disrupts protein synthesis by efficiently binding to the 70S ribosome [75]. Exploring the antibacterial mechanism of an AMP crustin, MjCru I-1, revealed its ability to bind bacterial cell wall molecules, such as LPS, LTA, and PG. Artificially synthesized whey acidic protein (WAP) domain of the crustin showed higher antibacterial and agglutination activity when compared to the cysteine-rich domain [76]. A study indicated that the WAP domain of the AMP crustin, plays an important role in antimicrobial activity against Gram-negative and Gram-positive pathogens by permeabilizing their membranes [77]. Further, the role of AMPs as defense peptides in immunomodulation of immune responses was revealed**.** In addition to their direct antimicrobial and antiviral activity, shrimp AMPs can also modulate the immune system [78]. Crustins in particular, function as opsonins, attaching to pathogen surfaces, coating pathogens, and enhancing their recognition for phagocytic uptake by hemocytes [79].

**Fig. 2:**
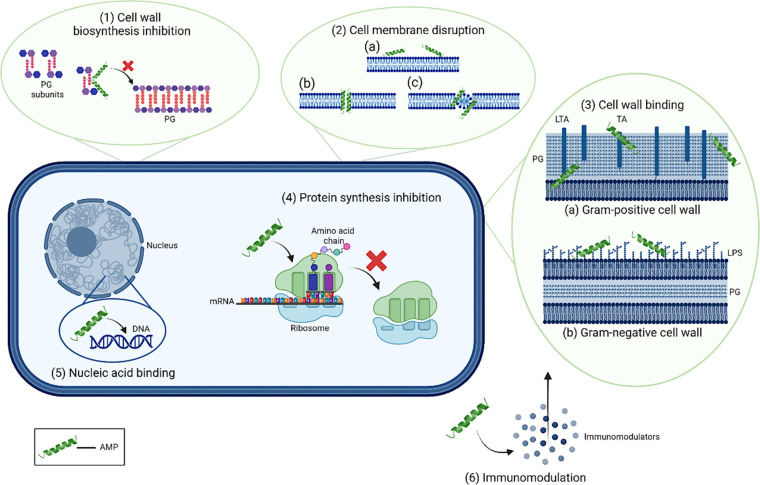
Mechanisms of action of AMPs on bacteria: (1) Cell wall biosynthesis inhibition: AMPs disrupt the cell wall formation in bacteria by targeting and binding to peptidoglycan (PG) subunits, thereby preventing synthesis of the intact cell wall and weakening the bacterial cell integrity. (2) Cell membrane disruption: AMPs are capable of permeabilizing the cell membrane through various mechanisms; (a) Carpet model where AMPs attach themselves to the bacterial membrane, destabilizing the integrity of the phospholipid layer; (b) Barrel stave model where AMPs bind to the surface and insert themselves into the membrane, causing the formation of a pore, resulting in expulsion of intracellular contents; (c) Toroidal pore model where AMPs attach to the surface, embed into the cell membrane and form a ring pore that results in efflux of ions and smaller molecules. (3) Cell wall binding: (a) In Gram-positive bacteria, AMPs show the ability to bind to teichoic acids (TA), lipoteichoic acids (LTA), and peptidoglycan (PG). (b) In Gram-negative bacteria, AMPs bind to the lipopolysaccharide (LPS) and PG. (4) Protein synthesis inhibition: AMPs interfere with the ribosome functionality, interrupting the translation process and halting protein synthesis. (5) Nucleic acid binding: AMPs enter bacterial cells and bind to the nucleic acids, disrupting DNA and RNA functions. (6) Immunomodulation: Alongside direct antimicrobial properties, AMPs can also act as immunomodulators by recruiting immune cells and enhancing the host immune responses against various pathogens.

Additionally, studies have shown the antifungal properties of shrimp-derived stylicin AMP, owing to the action of three cysteine residues in the peptide, which confer immunomodulatory effects [80]. In yet another investigation of the action of shrimp ALF, it was observed that shrimp ALF suppresses cytokine secretion by inhibiting the p38 and NF-κB pathways [81]. Such data suggest that, beyond their role as effector molecules, AMPs actively modulate immune signalling pathways and cellular responses during pathogen exposure. Upon supplementation of cell culture media with these peptides, carcinoma cell growth was inhibited, suggesting its antitumor activity [82]. A depiction of various mechanisms by which AMPs act in a bacterial cell is shown in Fig. 2.

### Classification of shrimp AMPs

Shrimp AMPs constitute a highly diverse class of immune effectors that control a wide range of pathogens. These peptides are effective in mediating antimicrobial activity and maintaining immune homeostasis. The majority of these AMPs carry a net positive charge, enabling them to bind to microbial membranes via electrostatic attraction. At the same time, their hydrophobic regions enable insertion into bilayers, ultimately leading to pathogen elimination [60,83]. AMPs are predominantly synthesized and stored in hemocytes, and are secreted into the hemolymph upon exposure to pathogens [58]. Several shrimp AMP families have been identified, including penaeidins, crustins, stylicins, and lysozymes [84]. The following sections describe the major classes of shrimp AMPs, highlighting their characteristics and antimicrobial functions.

#### Penaeidins

A family of AMPs that were first isolated from *P. vannamei* [85]. They are positively charged molecules with a unique N-terminal proline-rich region and a C-terminal cysteine-rich region containing three disulfide bonds. Penaeidins are classified into subgroups, namely PEN2, PEN3, PEN4, and PEN5 based on the position of certain amino acids and their sequences [86,87]. These AMPs are amphipathic, owing to the cysteine-rich domain that contains more positively charged amino acids than the proline-rich domains, suggesting that the cysteine-rich region plays a major role in pathogen recognition [88]. Additionally, the cysteine motifs in the C-terminal region are responsible for chitin binding and antibacterial activity [89]. Penaeidins possess potent antiviral properties by blocking viral entry into host cells through interactions with viral structural proteins. Functional studies using RNA interference (RNAi) knockdown of penaeidins in *P. vannamei* have revealed marked increase in the viral load and burdens in survival rates of the WSSV-challenged shrimp. The restoration of shrimp survival upon treatment with recombinant penaeidin proteins further confirms their antiviral response [90]. Additionally, they have been characterized for their antimicrobial activity against Gram-positive bacteria, with antimicrobial assays demonstrating inhibition of species such as *Micrococcus luteus, Bacillus epiphytes*, and *Staphylococcus haemolyticus*, with minimum inhibitory concentration (MIC) values as low as 0.2 μM against B*.* epiphyt*e*s. However, their activity against Gram-negative bacteria is somewhat limited, as Pen4–1 AMP has shown inhibition of only *E. coli* at higher MICs of 25–50 μM [91,92]. In addition to their antimicrobial activity, penaeidins are crucial for wound healing [48]. The reported MIC values of FmPEN3 (>50 μM) and FmPEN5 (50 μM) against *V. parahaemolyticus* are considerably higher for Gram-positive bacteria, suggesting comparatively weak efficacy against *Vibrio* species [93].

#### ALFs

Another family of AMPs, the ALFs, were first reported as an anticoagulant factor in chelicerates. Ever since the discovery, reports of ALF identification in diverse crustaceans have been on the rise, including in freshwater prawns, crayfish, crabs, and shrimps such as *P. monodon, P. vannamei*, and *Marsupenaeus japonicus* [94]. ALFs, with a sequence length exceeding 100 amino acid residues, contain a hydrophobic N-terminal signal peptide and a functional LPS-binding domain that facilitates binding to the cell wall of target bacteria [95]. The core domain of the ALF is the central β-hairpin, which is responsible for its biological activity. The mechanism of action of ALF lies in their ability to bind to LTA in Gram-positive bacteria, LPS in Gram-negative bacteria, and β-glucans in fungi. Evidence from functional studies has validated shrimp ALFs as potent AMPs with notable activity against Gram-negative *Vibrio* species. In *Fenneropenaeus chinensis*, FcALF8 and its synthetic LBD8 peptide have effectively inhibited *V. alginolyticus, V. harveyi*, and other pathogenic *Vibrios*. In addition, ALFs exhibit inhibitory activity against Gram-positive bacteria, including *Bacillus megaterium, Aerococcus viridans*, and *M. luteus* [74,96]. The ALF family of AMPs is primarily divided into seven groups with unique biochemical properties. Anion groups are A, D, E, and G, while B, C, and F are the cationic groups. Groups B and G show high degree of antimicrobial activity, groups A, C, and E have a limited range, while groups D and F show very weak antimicrobial activity [83]. Among shrimp ALFs, group B peptides demonstrate the highest antibacterial potency, with MIC values reaching 0.15 μM against both *B. megaterium* and *E. coli* and retaining their activity in the β-hairpin synthetic forms alongside the group G peptides like ALF-G34–55, which also displayed high activity against various Gram-positive (*Bacillus cereus*) and Gram-negative bacteria (*Vibrio nigripulchritudo*). Group A ALFs, although sometimes anionic, strongly retain the conserved β-hairpin domains, suggesting that further studies need to be carried out to determine if their activity depends on conserved amino acids or their charge. Group C ALFs remain cationic, whereas Group E ALFs are immune-responsive, with LPS-binding domains of MjALF-E1/E2 exhibiting strong LPS and PGN interaction, despite the weaker activity of recombinant MjALF-E2 peptide. In comparison, group D and F ALFs are composed of highly anionic peptides, which show negligible activity, with no detectable effects at MIC concentrations of 40 μM [74,97,98].

#### Crustins

These are an important family of AMPs, first identified in *Carcinus maenas* hemocytes and initially designated as carcinin. Later, other crustins were reported in various crustaceans, including crayfish, lobsters, crabs, and shrimp. Crustins possess various biological activities, including regulation of immune responses, broad-spectrum antimicrobial properties, and protease-inhibitory activity [99]. Crustins are typically characterized by a single WAP region at the C-terminal [100]. The WAP region forms a four-disulfide bond core at the C-terminal of the protein and is known to be linked with a range of functions. Also, the amino acid polytropic region residing between the signal peptide and the WAP region is pivotal for crustin functionality [79,101]. A recent study suggests that a newly identified type VI crustin in *P. vannamei* contributes to bacteria inhibition by binding to polysaccharides, and enhancing phagocytosis [102]. Numerous attempts have been made to classify crustins systematically. The three-type classification for crustins was originally proposed. Later, the five-type classification system was introduced. In the latest development, the seven-type classification has been implemented based on structural characteristics and phylogeny analysis, of which class V is present only in insects [86,101].

#### Stylicin

This family of AMPs has been identified only in shrimp and bivalves, and their prevalence is very limited when compared to other AMP families. Structurally, they possess an N-terminal proline-rich region and a C-terminal with 13 cysteine residues [58]. Identified in *Litopenaeus stylirostris*, Ls-stylicin1 is the initial member of the emerging stylicin family that predominantly targets fungi. Based on the relatively high MIC, it was hypothesized that the establishment of direct membrane disruption was improbable, suggesting that the mechanism of bacterial growth inhibition could depend on the ability of rLs-stylicin1 to bind to the cell walls [103]. Recent research has uncovered a novel stylicin Mi-sty isolated from the Kuruma shrimp *M. japonicus* [104].

Apart from AMPs described above, many AMPs in crustaceans are sequestrated in larger proteins. Hemocyanins are oxygen-carrying proteins that are found in crustaceans, and other arthropods. In response to infections, hemocyanins in crustaceans release histidine-rich AMPs, which can mitigate various microbes across a broad range of mechanisms. Effective inhibition of various Gram-negative and Gram-positive bacteria has been exhibited by 20 AMPs discovered from hemocyanin of *P. vannamei* [52]. Hemocyanins exhibit high structural stability and bioactivity, making them suitable for strategic optimization in antimicrobial research [105]. In a recent study, PvL1, a hemocyanin-derived AMP has been shown to have potent antimicrobial activity against *V. parahaemolyticus* AHPND, both *in-vitro* and *in-vivo*. Further, it was shown that it also balanced the disrupted microbiota in AHPND-affected shrimp hepatopancreas [106]. PvHCt, a histidine-rich AMP from shrimp hemocyanin, predominantly shows antifungal activity by permeabilizing fungal cells [107]. Isolated from *V. parahaemolyticus-*infected *P. vannamei* sera, a novel histone-derived peptide, PvH4a, was observed to successfully prevent the growth of Gram-negative bacteria by disrupting cell walls [108]. A phosphatase-like protein (PLP) identified from *P. monodon* has shown inhibitory effects predominantly against *V. harveyi* and other Gram-negative and Gram-positive bacteria [109]. Leucine-rich repeat (LRR) domains are transmembrane proteins involved in immune responses. In a recent study, the functional knockdown of the LLR domain LvLRRm1 in shrimp resulted in increased susceptibility to *V. parahaemolyticus* infection [110]. Another newly identified peptide, Litopeidin_28–51_, has shown substantial direct antiviral activity against WSSV by inhibiting the expression of key viral genes, such as *IE1* and *VP28* [111].

## Action of AMPs on shrimp pathogens and their expression pattern

AMPs are an important component in the innate immune system that plays a major role in shrimp defense against microbial invasion [112]. In general, the cellular and humoral immune response are responsible for eliminating the pathogens. As part of cellular responses, phagocytosis, and encapsulation are initiated by the PRPs, whereas on the humoral response front, the proPO system, blood clotting, and AMPs play the part [62]. AMPs, the soluble molecules in the humoral immunity pathway, safeguard shrimp against various pathogens [113]. The quick and effective response elicited makes them the first line of defense especially in marine invertebrates [114]. In penaeid shrimp, they form a part of the immune system targeting a range of Gram-negative and Gram-positive bacterial pathogens, yeasts, and viruses [62]. Infections are combated by AMPs that are secreted by a general mechanism that involves the PRPs to bind to PAMPs, leading to the activation of immune signalling cascades [115]. In *P. monodon*, ALFPm3 inhibited the growth of various fungi and Gram-positive and Gram-negative bacteria, including the most threatening shrimp pathogens, *V. harveyi* and *V. parahaemolyticus*. Recombinant ALFPm3 was shown to cause bacterial lysis by permeabilizing the membrane [116]. Another AMP, ALFPm10 from *P. monodon*, was found to be effective against aquaculture pathogens including *Aeromonas hydrophila, Streptococcus agalactiae*, and *V. parahaemolyticus* within 12 h of infection [117]. A knockdown study using an ALF belonging to group E, named MjALF-E2, isolated from *M. japonicus*, revealed a significantly higher mortality rate and increased susceptibility to *Vibrio anguillarum,* suggesting its role as part of the immune protection offered in shrimp [118]. A study analyzing the antimicrobial and immunomodulatory effects of a recombinant crustin isolated from deep-sea shrimp of *Rimicaris* sp*.* found that the recombinant crustin effectively killed Gram-positive and Gram-negative bacteria [78]. CarcininPm1 and CarcininPm2 are predominantly expressed AMPs in shrimp intestine and hemocytes, and their expression correlates with rapid increase in hemocyte production during early stage of bacterial infection [119]. AMPs have been found to inhibit biofilm formation, a common mechanism for development of AMR in bacterial pathogens [120]. The antibiofilm and antibacterial effectiveness have been validated by concentration-dependent *in-vitro* testing. In a study, crustin isolated from *Penaeus semisulcatus* was shown to possess antimicrobial activity against various Gram-negative and Gram-positive bacteria and antibiofilm activity, with a maximal effect at 40 µg/ml after 24 h [121]. Similarly, calcineurin A, a peptide isolated from *Macrobrachium rosenbergii*, showed antibacterial and antibiofilm effects against a wide range of shrimp pathogens, including *V. harveyi*. Treatment with its derived peptide VV18 disrupted the biofilm matrix of *E. coli* ATCC 9637, breaking intercellular connections, separating cells, and altering their morphology, as confirmed by SEM imaging [122]. Another AMP, haemocyanin (Hc) from *P. semisulcatus* showed potent antibacterial and antibiofilm activities against many Gram-positive and Gram-negative bacteria. Purified Hc reduced biofilm formation by 71 – 95 % in major shrimp pathogens at 50 – 100 µg/ml. Furthermore, docking studies that revealed strong interaction between Hc and bacterial ligands proposed Hc as a potential drug candidate in the aquaculture sector [123]. Recombinant stylicin, another AMP, was found to inhibit *V. parahaemolyticus* biofilm formation at 200 µg/ml, thus favouring its role in potentially enhancing shrimp survival post-infection [124].

Studies on the expression patterns of AMPs in different tissues in response to various microbial challenges revealed predominantly upregulation in haemocytes. Varied expression patterns were also observed in other tissues, such as the gills and hepatopancreas, suggesting their roles in AMP production [125]. In shrimp, haemocytes are the primary site of AMP expression and remain the main source of triggering the immune response by migrating to infection sites to combat invaders [60]. Hemocytes thus contribute to both cellular and humoral immunity by participating in various immune functions, such as phagocytosis, melanisation, coagulation, and AMP production. Crustacean immunity is divided into four types based on responses to infections caused by pathogens such as WSSV: semi-granular, granular, proliferation-associated, and immunity-activated [126]. Penaeidins are AMPs that comprise 30–40 % of the secreted AMPs expressed in naive and untreated shrimp in their active form in the granular hemocytes. Primarily stored in the cytoplasmic granules of larger granular hemocytes, they are also stored in the smaller granules to some extent [127]. In shrimp, immune responses are characterized by extensive hemocyte recruitment to infection sites, with penaeidin aiding in eliminating bacteria. *In-vivo* studies following injection of heat-killed pathogens demonstrated a major migration of hemocytes and lysis, with a proliferative recovery phase [128]. The distribution, expression profiles, and function of AMPs in shrimp differ according to the AMP type [129]. Expression of type IIa crustins in penaeid shrimp mainly occurs in hemocytes. Investigation of crusFpau, a type IIa crustin isolated from *Farfantepenaeus paulensis*, showed its synthesis and storage in both granular and semigranular hemocytes [130]. Expression of another AMP Fi-PEN3 was predominantly observed in hemocytes, with sparse presence in hepatopancreas. Transcripts of *Fenneropenaeus indicus* were abundant in hemocytes and present in intermediate levels in muscles, gills, heart, and intestine. This suggests the distinct mechanisms and pathways that control AMP gene activity and expression in shrimp tissues [131]. In a transcriptomic study of shrimp humoral immunity, five AMP genes showed significant upregulation in response to *V. parahaemolyticus* infection. The genes expressed in six tissues showed increased expression levels in response to both Gram-negative and Gram-positive bacteria [132]. AMPs in shrimp exhibit varied functionality across species, with marked differences in expression dynamics, regulation, and antimicrobial performance within hosts. Comparative analysis of penaeidins, designated penaeidin 4 from *P. vannamei* and *Litopenaeus setiferus*, revealed substantial diversity in the peptide's mature domain sequences, with striking variability in their functional segments [133]. Another study has revealed that sequence comparisons reveal differences in key features in the WAP domain of Type IIa and Type IIb crustins, originating from a common ancestral gene across shrimp species, including *P. vannamei, P. monodon, F. chinensis*, and *M. japonicus*. Type IIb crustins within a single species are diverse, reflecting distinct evolutionary pressures and possible functional specializations [134]. Differences in AMP sequence diversity and isoform composition have been linked to variation in antimicrobial specificities and differential immune responsiveness during infection by pathogens. Table 1 lists details on constitutive variations in expression levels across tissues and on challenge studies of AMPs.

**Table 1:** Shrimp AMP expression analysis in various tissues and challenge studies.

AMP	Shrimp species	Tissue type- high expression (Constitutive)	Tissue type- low expression (Constitutive)	Challenge study	References
Type I crustin	*Marsupenaeus japonicus*	Gills, stomach	Hemocytes, heart, hepatopancreas, intestine	*V. anguillarum* and *Staphylococcus aureus*	[76]
Anti-lipopolysaccharide factor (ALF)	*Fenneropenaeus chinensis*	Lymph organ (Oka)	Muscle, intestines, stomach, hepatopancreas, hemocyte, heart, nerve, gill, eyestalk	*Micrococcus lysodeikticus* and *V. anguillarum*	[135]
Lysozyme	*M. japonicus*	Hepatopancreas	Gills, stomach, muscle, heart, eyestalk, intestine, hemocyte	White spot syndrome virus (WSSV)	[136]
Crustin	*Penaeus vannamei*	Gills, hemocytes, epithelia, eyestalks	Hepatopancreas	*Vibrio parahaemolyticus* and WSSV	[137]
Class III Penaeidin	*F. chinensis*	Hemocytes, gills, heart. Intestine	Hepatopancreas, eyes, subcuticular epithelia, brain, stomach	-	[138]
ALF	*P. vannamei*	Lymphoid organ	Ovary, testis	*V. parahaemolyticus* and WSSV	[139]
Stylicin	*P. vannamei*	Circulating hemocytes, foregut, midgut, hindgut, gills, nerve cord	-	*Vibrio harveyi* and WSSV	[140]
Penaeidin	*P. vannamei*	Hemocytes, heart, gills, epigastric haematopoetic nodules, testis	Eyes, intestine, subcuticular epithelium, hepatopancreas	*Aerococcus viridans, V. alginolyticus, Fusarium oxysporum*	[141,142]
Penaeidin	*Fenneropenaeus indicus*	Hemocytes, heart, gills, muscles	Hepatopancreas, eyestalk, intestine	*V. parahaemolyticus*	[143]
Stylicin	*M. japonicus*	Gills, hemocytes	Hepatopancreas, stomach, heart, muscle, eyestalk, intestine	WSSV	[144]
ALF	*P. vannamei*	Lymphoid organ, heart, gills, eyestalk, hemocytes	Muscle, hepatopancreas	*Vibrio penaeicida, F. oxysporum*, WSSV	[145]
Crustin	*Pandalopsis japonica*	Gills, epidermis	Hemocytes, brain, abdominal ganglia, thoracic ganglia, flexor muscle, extensor muscle, heart, gonad, hemocyte	-	[146]
ALF	*P. vannamei*	Hemolymph, gills, muscles	Hepatopancreas, intestine	WSSV	[147]
Penaeidin	*Penaeus monodon*	Hemocytes, neural ganglian, ovary, gills, intestine, mandibular organ	Heart, eyestalk, hepatopancreas, muscle, subcuticular epithelium	-	[148]
Lysozyme	*P. vannamei*	Hemolymph	Brain, eyestalk, hepatopancreas, heart, stomach, gills, intestine, muscle	*V. harveyi*	[149]
Lysozyme	*P. vannamei*	Hemocytes, heart, lymphoid organ, and muscle	Gills	*Vibrio campbelli*	[150]
Crustin (Mn-Gly-Cru1)	*Macrobrachium nipponense*	Gills	Hemocytes, heart, hepatopancreas, stomach, intestine	WSSV, *V. parahaemolyticus, S. aureus*	[151]
ALF	*P. monodon*	Stomach and lymphoid organs	Muscle, hemocyte, hepatopancreas, hind gut, heart, pleopod, and gills	*V. parahaemolyticus*	[152]
Crustin- like	*M. japonicus*	Hemocytes	-	*-*	[153]
ALF-like	*F. chinensis*	Hemocytes, gills, and intestine	Hepatopancreas	*V. anguillarum*	[154]

Note: (-), no information available.

## *In silico* analysis and recombinant production of shrimp AMPs

Computational analysis and *in silico* tools have emerged as a powerful platform for detecting and characterizing shrimp AMPs from extensive transcriptomic and genomic databases. High-throughput sequencing and omics technologies, such as proteomics and transcriptomics, have further enabled rapid identification of new AMPs across diverse species [155]. The emergence of artificial intelligence (AI) and machine learning (ML) has advanced as tool for the design and discovery of AMPs, enabling the prediction of activity, 3D modelling, and rational sequence optimization, which can accelerate the translation of sequences into effective bioactive molecules [156]. Genomic and transcriptomic analyses in *P. vannamei* have revealed 754 genes encoding AMPs across 24 distinct families. 20 chemically synthesized peptides demonstrated antimicrobial activity of more than 85 % against major shrimp pathogens, including *S. aureus* and *V. parahaemolyticus* [157].

After identification, the structural and physicochemical properties of the peptides can be examined**.** For instance, a mere structural assessment of Pmthymosin3 in *P. monodon* which revealed an α-helical conformation, suggested its possible antimicrobial activity. Further functional studies highlighted its tissue-specific expression patterns and antimicrobial properties, reinforcing its value as a candidate AMP for further research [158]. Although *in silico* methods can speed up the discovery of AMPs, recombinant DNA (rDNA) technology offers a more practical approach for large-scale production of AMPs.

Host defense peptides (HDPs) are synthesized by automated solid-phase peptide synthesis (SPPS) and are widely used. However, despite its widespread use, the high production costs and environmental concerns associated with solvent consumption limit its applicability and scalability. The commercial production of HDPs using SPPS presents environmental challenges. The process mainly relies on toxic organic solvents, such as dimethylformamide (DMF) and dichloromethane (DCM), which generate significant solvent waste due to the repeated reaction steps and coupling procedures [159]. As an alternate strategy, rDNA technologies have emerged as a promising option for HDP production [160]. While direct isolation and purification of AMPs is possible, the labor-intensive process yields insufficient amounts, posing significant challenges for cost-effective large-scale production. In contrast, rDNA technology offers a viable alternative for industrial-scale AMP production using genetically modified organisms such as yeasts and bacteria [161]. Such microbial expression systems using eukaryotic and prokaryotic hosts have been explored to achieve mass production at lower costs. A modified FcALF2 (mFcALF2) gene from *F. chinensis* was designed and synthesized with an LPS-binding domain substitution, and its expression was carried out in *Pichia pastoris*, which, when tested, showed significant antibacterial effects against various Gram-negative and Gram-positive bacteria [162]. AMPs, characterized by their high positive charge and low molecular weight, are inherently bactericidal but prone to proteolytic degradation. They may exhibit toxicity against eukaryotic and prokaryotic cells. To overcome this, the strategic selection of host organisms for efficient production of functional recombinant AMPs with improved expression yields becomes critical [161]. The recombinant production technology enables investigation of their structure, composition, function, and mechanism of action. It provides for large-scale industrial applications by facilitating scalable, sustainable, and cost-effective production compared to purification from natural sources and chemical synthesis [163]. rDNA technology is superior to the existing traditional methods for producing AMPs due to their structural complexity, like disulfide bonds within the functional WAP domain in the case of crustin. Recombinant crustin has been successfully expressed in both eukaryotic (*P. pastoris*) and prokaryotic (*E. coli*) systems [164]. Recombinant expression enables site-directed mutagenesis, facilitating exploration of the roles of antibacterial residues and supporting diverse biotechnological applications. Lysozyme of *P. vannamei* has been overexpressed in *E. coli*, with sufficient amount of the protein being obtained, thus rendering it useful for functional and biochemical characterization [165]. A crustin isoform from *P. monodon* has been cloned and expressed in *E. coli*, showing a strong bactericidal effect against various Gram-negative and Gram-positive bacteria, particularly shrimp pathogens. The peptide's low MIC against *Vibrio* pathogens underscores its potential in shrimp aquaculture [166]. Researchers have cloned a cDNA encoding MjALF2, a novel ALF from *M. japonicus*. Elevated expression of MjALF2 in lymphoid organ cells was observed after LPS stimulation, indicating its role in defense and further expanding its use for disease control [167]. Recombinant shrimp AMPs are increasingly being recognized for their potential application in shrimp health management and disease control. A recombinant shrimp c-type lysozyme (rLvLyz-c) in *P. vannamei* supplemented in the diet enhanced survival, upregulated immune-related genes and improved growth [168]. Based on the outcomes of recombinant expression of shrimp AMPs, it can be inferred that rDNA technology is highly beneficial. These peptides show strong antimicrobial activity against major shrimp pathogens, offering sustainable disease control and enhanced productivity. Table 2 lists recombinantly produced shrimp-derived AMPs and their antimicrobial efficacy in various expression systems.

**Table 2:** Recombinant production of shrimp-derived AMPs, expression systems, and antimicrobial spectrum.

Shrimp species	AMP name	Vector	Host	Protein size	Antimicrobial spectrum	Reference
*Fenneropenaeus indicus*	Lysozyme	pET – 28a expression vector	*E. coli* BL21 cells	20 kDa	*Salmonella T*yphimurium*, E. coli, Pichia pastoris*	[169]
*Marsupenaeus japonicus*	ALF	pET – 32a expression vector	*E. coli* Rosetta cells	-	White spot syndrome virus (WSSV)	[94]
*Penaeus monodon*	Crustin	pET – 28a expression vector	*E. coli* BL21 cells	14.7 kDa	*Bacillus megaterium, Micrococcus luteus, Streptococcus iniae, S. aureus, Aerococcus viridans, Staphylococcus haemolyticus, E. coli*	[170]
*Fenneropenaeus chinensis*	ALF	pMD19-T cloning vector, pET30a expression vector	*E. coli* BL21 cells	15 kDa	*V. alginolyticus, Bacillus subtilis, M. luteus*	[171]
*F. chinensis*	Penaeidin	pPIC9K expression vector	*P. pastoris* KM71 cells	7 kDa	*S. aureus, M. luteus, Bacillus thuringiensis, Bacillus cereus, B. subtilis, B. megaterium, E. coli, Klebsiella pneumonia, Fusarium solani, Gloeosporium dahlia, Fusarium oxysporum, Colletotrichum lagenarium*	[172]
*M. japonicus*	Lysozyme	pET – 32a expression vector	*E. coli* BL21 cells	34 kDa	*Micrococcus lysodeikticus, S. aureus*	[173]
*F. chinensis*	Crustin	pET – 30a expression vector	*E. coli* BL21 – DE3 cells	22 kDa	*S. aureus, B. thuringiensis, B. subtilis, B. cereus, B. megaterium*	[174]
*F. chinensis*	Crustin-like protein	pMD18-T cloning vector, pPIC9K expression vector	*P. pastoris* KM71 cells	13 kDa	*S. aureus*	[175]
*Penaeus vannamei*	Crustin	pEASY-T1 cloning vector, pYE-GAPα expression vector	*P. pastoris* GS115 cells	19.9 kDa	*S. aureus, B. subtilis, E. coli, V. parahaemolyticus*	[176]
*P. monodon*	Lysozyme	pBV220 expression vector	*E. coli* DH5α cells	16.32 kDa	*Aeromonas hydrophila, Edwardsiella tarda*¸, *V. anguillarum, V. alginolyticus, V. parahaemolyticus, E. coli, M. lysodeikticus*	[177]
*M. japonicus*	ALF	pET – 32a expression vector	*E. coli* Rosetta DE3 cells	-	*V. anguillarum, E. coli, S. aureus, B. megaterium, B. subtilis, B. thuringiensis*	[178]
*P. vannamei*	Penaeidin	pCRscript SK (+) cloning vector, pTG4812 expression vector	*Saccharomyces cerevisiae* TGY 48–1	5.5 kDa and 6.6 kDa	*M. luteus, E. coli, F. oxysporum, A. viridans, B. megaterium, Nectria haematococca, Neurospora crassa, Alternaria brassicola, Botrytis cinerea*	[179]
*F. chinensis*	Lysozyme	pET – 30a expression vector	*E. coli* BL21 – DE3 cells	27 kDa	*S. aureus, M. luteus, B. thuringiensis, B. cereus, B. subtilis, B. megaterium, K. pneumoniae*	[180]
*P. monodon*	ALF	pPIC9K expression vector	*P. pastoris* KM71 cells	11.31 kDa	*A. viridans, B. megaterium, M. luteus, Enterobacter cloacae, Erwinia carotovora, E. coli, K. pneumoniae, S. T*yphimurium*, V. anguillarum, V. alginolyticus, Vibrio harveyi, F. oxysporum, B. cinerea, Penicillium crustosum*	[181]
*P. monodon*	Crustin	pGEM-T Easy cloning vector, pET–19 b expression vector	*E. coli* BL21 – DE3 cells	17 kDa	*S. aureus, M. luteus, S. haemolyticus*	[182]
*F. chinensis*	Crustin-like	pMD18-T cloning vector, pCR® T7/NT TOPO® expression vector	*E. coli* BL21 – DE3 cells	11.5 kDa	*S. aureus, B. subtilis, B. megaterium, B. cereus, M. luteus*	[183]
*P. monodon*	Crustin-like	pGEM-T Easy cloning vector, pET–28 b expression vector	*E. coli* BL21 – DE3 cells	12.8 kDa	*S. aureus, B. megaterium, V. harveyi, S. haemolyticus, K. pneumoniae, E. coli, M. luteus, A. viridans*	[184]
*P. monodon*	Penaeidin	pUC-T cloning vector, pABhRpX expression vector	Sf21 insect cells	6.1 kDa	*A. viridans*	[185]
*P. vannamei*	ALF	pGEM-T Easy cloning vector, pMT/BiP/V5-His C Drosophila expression vector	Drosophila S2 cells	16.4 kDa	*E. coli, Bacillus amyloliquefaciens, V. parahaemolyticus* (suggested antimicrobial spectrum)	[186]
*P. monodon*	Lysozyme	pET-19b expression vector	*E. coli* Rosetta DE3 cells	15.5, 14.4, and 16.5 kDa	*M. luteus, V. harveyi, V. alginolyticus, Vibrio cholerae, V. parahaemolyticus,* and *Vibrio fluvialis*	[187]
*P. monodon*	Lysozyme	p EXP5-NT/TOPO TA expression vector	*E. coli* BL21 cells	20 kDa	*V. harveyi, E. coli, Listeria monocytogenes, S. T*yphimurium*, S. aureus, V. alginolyticus, V. parahaemolyticus, V. vulnificus, V. cholerae, Vibrio fischeri*, and *M. luteus.*	[188]

Note: (-), no information available.

Alongside computational screening and recombinant expression studies, *in-vivo* verification remains crucial to confirm the protective potential of shrimp AMPs. Studies demonstrate that recombinant LvCrustinB improves host survival against *V. parahaemolyticus* infection by virtue of its strong bacterial-binding capacity [176]. Another recombinant AMP, rALFPm3, has successfully inhibited the growth of *V. harveyi* and reduced shrimp mortality when administered as a prophylactic agent [189]. However, challenges such as rapid proteolytic degradation and non-specific delivery hinder their direct *in-vivo* application, underscoring the importance of nanotechnology-based strategies and feed-based approaches to enhance stability, bioavailability, and applicability [190].

## Limitations and knowledge gap

Shrimp AMPs have emerged as highly promising candidates for enhancing disease management and the overall health sustainability in shrimp aquaculture [48]. Although remarkable progress has been made and numerous studies have convincingly demonstrated their strong antimicrobial activity, immunomodulatory potential, and functional relevance, this field continues to evolve, with considerable scope for further scientific exploration. Elaborating on the current knowledge through large-scale, field-based testing would greatly enhance confidence in their practical applicability and translational potential within modern aquaculture practices [9,11]. Several AMPs in development suffer from poor stability, fast degradation, and delivery limitations, which have posed as a bottleneck for further application, highlighting the need for enhanced delivery strategies. Additionally, for the application of peptides in water-based systems at the field level, several important aspects, such as peptide stability, interactions with environmental disease parameters, host physiological responses, and the overall delivery efficiency of AMPs, can be emerging areas of investigation [53,69]. Although AI and ML approaches are increasingly used for AMP design and discovery, their practical effectiveness remains hindered by limited negative AMP sequences in databases, which limit accurate predictions [191]. Recombinant expression of AMPs and their nanodelivery strategies have shown significant progress in recent years. However, further improvements in peptide function efficiency, *in-vivo* stability, and bioavailability would greatly contribute to ongoing efforts to enhance application [192]. From a policy and implementation perspective, advancing well-defined regulatory mechanisms and frameworks, standardized assessment protocols, and robust guidelines will be essential to ensuring that AMP-based therapeutics are adopted responsibly within aquaculture practices [31]. Thus, by addressing these regulatory and experimental gaps through continued efforts, shrimp AMPs can transition from *in-vitro* experimental practices to commercially implementable products that are widely applicable for future shrimp aquaculture development.

## Future scope: nanoformulation of AMPs and genetic engineering

Despite extensive efforts to optimize AMP production, challenges exist such as insufficient yield, AMP degradation, and activity loss. Using fusion tags mitigates the toxic effects of recombinant AMPs (rAMPs) and enhances their stability. However, the susceptibility to proteolytic degradation and its cytotoxicity remains a hurdle [193,194]. In bacterial systems, AMPs are commonly used with carrier proteins such as glutathione S-transferase (GST), thioredoxin, or the small ubiquitin-like modifier (SUMO), which enhance the stability and solubility of the AMP [195]. Antibiotic overuse has resulted in the emergence of multidrug-resistant (MDR) pathogens, including methicillin-resistant *Staphylococcus aureus* (MRSA), motivating the exploration of nano-enabled AMP delivery systems for effective management of shrimp aquaculture [196,197]. In human clinical trials of therapeutic AMPs, peptides showed minimal antibacterial activity, possibly due to the differences when present in their natural environments, where they may act synergistically with various other compounds in pathways. Similar barriers are expected in aquaculture applications, thereby supporting the need for nanoformulation and advanced delivery strategies to enhance their applicability. On the other hand, isolated synthetic AMPs lack this interaction, suggesting that nanoformulations could effectively enhance their clinical efficacy by re-establishing and mimicking the properties of their natural counterparts [198]. Peptide delivery with nanoparticles optimizes the therapeutic effect of AMPs by preventing proteolysis, enhancing site-specific targeting, and reducing toxicity. Developing advanced nanocarriers can boost these properties, causing less harm to normal tissues [199]. The required dose to combat MDR bacteria can also be lowered [198]. Nanotechnology offers significant advantages, including a high surface area-to-volume ratio, high specificity to the target area, surface charge alteration, and reduced adverse effects. Suppressed or modified recombinant proteins delivered to shrimp effectively induced an innate response against WSSV [200]. The use of nanomaterials in the size range of 1–100 nm has proven to be a perfect delivery system for AMP delivery. Various nanomaterials for AMP delivery, such as metallic (gold nanoparticles (AuNPs), silver nanoparticles (AgNPs), gold nanodots) and polymeric (poly(lactic-co-glycolic acid) (PLGA), chitosan) nanoparticles, have been studied [201]. Among these nanoparticles are inorganic nanoparticles (metal nanomaterials and carbon nanotubes) and other nanosystems, such as polymer- and lipid-based systems [202]. Two approaches to nanoencapsulation are active targeting, in which ligands guide AMPs to target sites, and passive targeting, in which the carriers maintain the surface properties of the AMPs [203]. Through controlled-release strategies, nanoAMPs extend their action duration, enhance their half-life, minimize potential toxic effects, and maintain biological activity through consistent dosing [204]. Seagrass *Cymodocea serrulata* derived AgNPs exhibited potent antibacterial activity against the shrimp pathogen *V. parahaemolyticus* and were suitable for mass production due to their cost-effectiveness and environmentally friendly green synthesis when compared to the chemically synthesized AgNPs [196]. Administration of AgNPs to *P. monodon* through shrimp feed restored normal hepatopancreatic function in the infected shrimp [205]. Nanoparticle carriers, such as chitosan, alginates, and PLGA, used to deliver vaccine antigens alongside orally administered inflammatory stimulants demonstrated significant protection for fish and shellfish, achieving a 85 % survival rate in shrimp aquaculture [206]. Chitosan tripolyphosphate (CS/TPP) nanoparticles were used to orally deliver the VP28 protein to shrimp, resulting in enhanced immune gene expression and immunological parameters. This nanoformulation rendered protection against WSSV in shrimp, indicating its potential as an effective and safe approach for protein delivery [207]. Nanoformulation of AMPs to increase their stability and ensure targeted delivery in shrimp offers immense benefits to the shrimp aquaculture sector, as it can prospectively improve shrimp immune defense against pathogens, with consequent improvements in survival rates. In shrimp aquaculture, the targeted delivery of AMPs using methods such as microencapsulation enhances stability and bioavailability and overcomes the susceptibility of peptides [201]. While nanotechnology offers various benefits, it is paramount to recognize potential issues, such as nanotoxicity in aquatic organisms arising from nanoparticle interactions and tissue accumulation, before field applications [208]. However, different delivery systems and approaches have practical limitations; for instance, nanocapsulation increases production costs and requires specialized technical expertise for large-scale applications. Other barriers may include varied polydispersity, storage stability, and operational feasibility, which can limit the adoption of this technology in commercial shrimp farming [209]. The rapid integration of nanotechnology into fisheries is ushering in tools such as rapid disease diagnostics and improved delivery mechanisms. In shrimp farming, these advancements promise to pave the way for economically viable and sustainable development [210].

Another technology to improve aquaculture productivity is altering genetic material by incorporating the DNA of interest into a different host. In aquaculture, the fish *Oncorhynchus mykiss* is one of the first genetically modified organisms (GMOs) [211]. This method can be used to improve traits such as disease resistance, enhanced growth, stress tolerance, and reproductive performance, and has already been shown to be useful in cultured fish, especially for improving disease resistance [212,213]. Such techniques facilitate improvements in functional genomics and therapeutics for various fish and crustaceans [214]. Newer, advanced techniques such as CRISPR/Cas9 gene-editing systems enable precise genomic modifications in shrimp and other cultured aquatic species to improve immune responses. A recent study used the CRISPR/Cas9 system to target the WSSV genome in *P. vannamei* [215]. A similar approach could be used to enable precise modification of shrimp AMP-encoding genes to improve resistance to disease. Viral vectors are also valuable as gene delivery tools. For instance, recombinant baculovirus vectors have transduced and expressed the green fluorescent protein (GFP) into *P. monodon* lymphoid cells [216]. Also, a lentivirus-mediated gene transfer system has been used to transduce genes into shrimp lymphoid cells [217]. Such experiments have validated the use of viral vectors as potent gene delivery tools for genetic engineering in shrimp. Genetic modification of AMPs in shrimp can improve immunity, thereby reducing antibiotic use and advancing shrimp aquaculture. However, it is to be borne in mind that genetic modification can cause adverse effects on environment [218]. Hence, before releasing modified shrimp into the environment, all risks must be thoroughly evaluated, and policies must be developed rather than restricting the application of genetic engineering [219].

Despite advances in genome editing, off-target effects and insufficient understanding of genes linked to key traits remain major barriers in aquaculture. These issues complicate and restrict large-scale adoption of genetic modification technology in farming systems, necessitating stringent biosafety assessment before commercial deployment in field applications. Many countries have established regulatory frameworks to guide the approval of genetically modified organisms for environmental release and commercial use. The core purpose of these guidelines is to reduce the risk of environmental contamination [220]. A study has successfully optimized a CRISPR/Cas9 microinjection system in *M. rosenbergii*. This allowed targeted editing of the *MrPAX6* and *MrIAG* genes, resulting in phenotypic impacts such as 50 % reduction in eye pigmentation. Such outcomes establish a genetic tool that contributes to aquaculture improvement, but studies addressing disease resistance remain scarce [221]. Collectively, these considerations emphasize that technical feasibility alone is insufficient; assessing associated risks, complying with regulations, and implementing strategies that are practically specific to the use of genetically modified organisms are equally important in aquaculture systems. Although direct evidence from shrimp-derived AMPs remains limited, Table 3 presents a comprehensive comparative overview of commonly used recombinant expression systems for AMP production and nanocarrier development, highlighting their advantages and practical limitations.

**Table 3:** Comparative evaluation of recombinant AMP expression systems and nanocarrier delivery platforms.

	Host / carrier type	Advantages	Limitations	Relevance to shrimp aquaculture	References
rAMP production	*Pichia pastoris*	Secretory expression and disulfide bond formation of proteins; scalable fermentation	Methanol acts as inducer, which at high levels, causes toxicity; requires extensive optimizations	Recombinant ALF (rALF) expressed and retained antibacterial activity; growth performance, disease resistance, immunity improved upon dietary supplementation in *Penaeus vannamei*	[222,223]
	*E. coli*	Low cost; high expression levels; fast growth; ease of genetic manipulation	Formation of inclusion bodies; protein misfolding; toxicity to host cells; codon bias	rALF-D from *Marsupenaeus japonicus* showed antiviral activity against white spot syndrome virus (WSSV), causing reduction of viral replication *in-vivo*	[94,224]
	*Saccharomyces cerevisiae*	Post-translational modifications and protein folding can be facilitated	Low yield; abnormal proteolytic activity and glycosylation	Penaeidins (Pen-2 and Pen-3a) showed antifungal and antibacterial activity against Gram-positive bacteria	[179,225]
Nanocarriers	Chitosan nanoparticles	Enhances the efficiency of encapsulation and facilitates controlled release; biocompatible	Variability in batch productions; poor solubility in alkaline and neutral	Chitosan nanoparticles were used to optimize the encapsulation of dsDNA for oral administration in shrimp; observed to provide over 70 % protection and controlled release, sharply reducing WSSV mortality.	[[226], [227], [228]]
	PLGA nanoparticles	Increased biocompatibility, enhanced sustained release, potential for oral delivery	Production involves toxic solvents; generates harmful residues	PLGA and chitosan-coated PLGA microspheres encapsulated viral binding protein PmRab7 for oral delivery in shrimp; facilitated sustained release, safe in-vivo, enhanced antiviral activity against WSSV.	[229,230]
	Alginate nanoparticles	Biocompatibility, low production cost, and targeted delivery due to pH-responsive gelation	Unstable, high burst release, poor encapsulation efficiency	Alginate microparticles encapsulated *Bacillus licheniformis* for oral delivery; improved survival rates through digestive conditions; targeted intestinal release; higher viable cell retention.	[[231], [232], [233]]

## Conclusion

Shrimp cultivation, a critical component of global aquaculture, has significant economic impacts. This compels the requirements for sustainable farming techniques to control and eradicate disease-causing pathogens. The indiscriminate use of antibiotics to control these pathogens has led to the emergence of antibiotic-resistant bacteria that pose a significant threat to One Health, comprising human, animal, plant, and environmental health. This underlines the urgent necessity for alternative strategies. Owing to their broad-spectrum activity and unique mechanisms, AMPs have demonstrated their versatility in combating infections and are promising alternatives to antibiotics, particularly in shrimp farming. Insightful perspectives can be gained by comprehending their classification and mode of action against shrimp bacterial pathogens. The role they play as part of the innate immune system of shrimp is dynamic, as seen from the results of research carried out on expression patterns of AMPs in response to challenges posed by pathogens.

Additionally, recombinant technology can produce AMPs for large-scale applications to ensure their stability, scalability, and other necessary properties. The future of the application of shrimp AMPs depends on adopting innovative strategies such as nanoformulation and genetic modification. Nanoformulation of AMPs can significantly improve their stability, specificity of action, bioavailability, and therapeutic efficacy. Incorporation of specific AMP genes into the shrimp genome to enhance disease resistance through genetic engineering can be an effective strategy to mitigate infections. This review covers progressive scientific strategies for transition from traditional antibiotics to AMPs in shrimp farming. A more resilient and sustainable shrimp aquaculture industry can be built by maximizing the effectiveness of AMPs and their integration into advanced biotechnology approaches.

## Funding

This study was partly supported by the Nitte (Deemed to be University) through the NUFR2 project (N/RG/NUFR2/NUCSER/2023/03). The authors acknowledge the support provided by the Department of IT, BT and S & T, Govt. of Karnataka in establishing the Centre of Excellence in Aquamarine Innovation at the institute for providing the resources and infrastructure for research in aquaculture.

## CRediT authorship contribution statement

**Belman Ananya:** Writing – original draft, Visualization, Methodology, Investigation, Formal analysis, Data curation, Conceptualization. **Vijay Gundmi Apurva:** Writing – review & editing, Software, Methodology, Formal analysis. **Indrani Karunasagar:** Writing – review & editing, Validation, Supervision. **Anirban Chakraborty:** Writing – review & editing, Validation, Supervision. **Biswajit Maiti:** Writing – review & editing, Validation, Supervision, Resources, Project administration, Investigation, Funding acquisition, Conceptualization.

## Declaration of competing interest

The authors declare that they have no known competing financial interests or personal relationships that could have appeared to influence the work reported in this paper.

## Data Availability

No data was used for the research described in the article.
